# Histamine and the striatum

**DOI:** 10.1016/j.neuropharm.2015.08.013

**Published:** 2016-07

**Authors:** J. Paul Bolam, Tommas J. Ellender

**Affiliations:** Department of Pharmacology, MRC Brain Network Dynamics Unit, Mansfield Road, OX1 3TH Oxford, United Kingdom

**Keywords:** Histamine, Striatum, Basal ganglia, Tourette's syndrome, Parkinson's disease

## Abstract

The neuromodulator histamine is released throughout the brain during periods of wakefulness. Combined with an abundant expression of histamine receptors, this suggests potential widespread histaminergic control of neural circuit activity. However, the effect of histamine on many of these circuits is unknown. In this review we will discuss recent evidence for histaminergic modulation of the basal ganglia circuitry, and specifically its main input nucleus; the striatum. Furthermore, we will discuss recent findings of histaminergic dysfunction in several basal ganglia disorders, including in Parkinson's disease and most prominently, in Tourette's syndrome, which has led to a resurgence of interest in this neuromodulator. Combined, these recent observations not only suggest a central role for histamine in modulating basal ganglia activity and behaviour, but also as a possible target in treating basal ganglia disorders.

This article is part of the Special Issue entitled ‘Histamine Receptors’.

## 1 Introduction

Both the hypothalamus and basal ganglia are evolutionary conserved brain structures essential for the survival of an organism. The hypothalamus is a small structure at the base of the brain important in controlling life-sustaining functions such as thermoregulation, metabolism and sleep and wakefulness, among others. In addition, it is thought that the hypothalamus is able to control the likelihood of more complex behavioural repertoires via its widespread projections throughout the brain (Saper and Lowell, 2014). The posterior part of the hypothalamus includes the tuberomamillary nucleus (TMN) containing histaminergic neurons that also project extensively and innervate nearly all regions of the brain (Watanabe et al., 1983, Panula et al., 1984). Whereas the activity of histaminergic neurons and the release of the neuromodulator histamine have been shown to be important for maintaining wakefulness and attention (White and Rumbold, 1988, Lin et al., 1989, Parmentier et al., 2002, Anaclet et al., 2009) increasing evidence suggests that histamine has wider functions in controlling behaviour and neural circuit function.

The basal ganglia are a large collection of subcortical nuclei, which play a central role in the control of motor behaviour, through the selection and recruitment of appropriate motor programs (Grillner et al., 2005), as well as cognitive function (Graybiel et al., 1994, Grillner et al., 2005, Yin and Knowlton, 2006). The basal ganglia receive a histaminergic projection (Takagi et al., 1986, Airaksinen and Panula, 1988) and in particular the major input nucleus of the basal ganglia, the striatum, expresses a high density of histamine receptors (Hill and Young, 1980, Martinez-Mir et al., 1990, Vizuete et al., 1997, Pillot et al., 2002) suggesting that histamine can directly affect striatal function and basal ganglia output.

This review will focus on the circuitry of the striatum, the role of histamine in modulating striatal synaptic transmission and behaviour and lastly the role of the histaminergic system in disorders of the basal ganglia including in Parkinson's disease and in Tourette's syndrome. We point to other excellent recent reviews (Haas and Panula, 2003, Haas et al., 2008, Panula and Nuutinen, 2013) and reviews in this special issue of Neuropharmacology for histaminergic modulation of sleep-wake states, molecular properties of histamine receptors and the expression profiles of specific histamine receptor subtypes and other neurological disorders linked to histamine dysfunction which we could not include for brevity.

## 2 The basal ganglia and the striatum

**Fig. 1:**
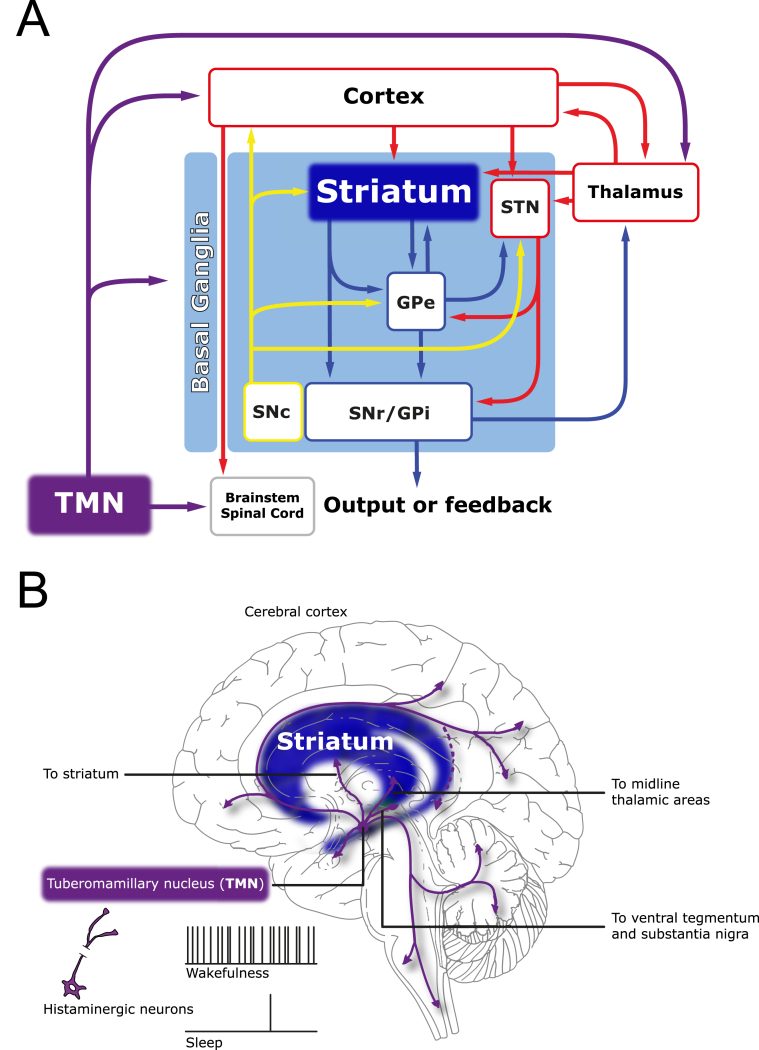
(A) A simplified, schematic diagram of the basal ganglia (within the pale blue box) and their associated structures. Glutamatergic connections are in red, GABAergic connections are in dark blue, dopaminergic connections are in yellow and histaminergic projections are in purple. The major excitatory afferents to the basal ganglia are from the cortex and thalamus and are directed to both the striatum and the *subthalamic nucleus* (STN). The striatum influences the basal ganglia output nuclei *substantia nigra pars reticulata* (SNr) and the internal segment of the *globus pallidus* (GPi) directly, or indirectly via connections with the network between the STN and external *globus pallidus* (GPe). The dopaminergic *substantia nigra pars compacta* (SNc) influences the operation of the basal ganglia via connections with each nucleus. Similarly, the histaminergic *tuberomamillary nucleus* (TMN) influences the basal ganglia as well as the input structures to the basal ganglia. The major targets of the basal ganglia output nuclei are the thalamus and the midbrain and brainstem premotor regions, which influence movement via direct or indirect connections with motor nuclei. (B) Histaminergic neurons located in the *tuberomamillary nucleus* project to nearly all regions of the brain. Emphasized here are the histaminergic projections to basal ganglia, in particular the striatum (blue), and the input structures to the basal ganglia, namely the cortex and thalamus. The histaminergic neurons fire at high frequency during wakefulness and are virtually silent during sleep.

The basal ganglia are an interconnected network of subcortical nuclei (Fig. 1A), which play a critical role in the control of motor behaviour and cognitive function (Graybiel et al., 1994, Grillner et al., 2005, Yin and Knowlton, 2006). The striatum is the main input nucleus of the basal ganglia, receiving and integrating cortical and thalamic excitatory glutamatergic input (Doig et al., 2010, Huerta-Ocampo et al., 2014) and exerts a strong control on downstream basal ganglia nuclei and behaviour.

The main neuronal type of the striatum, making up approximately 95% of all neurons in the striatum, is the GABAergic medium-spiny projection neuron (MSN), of which there are at least two classes; the dopamine receptor 1 (D_1_)-expressing and dopamine receptor 2 (D_2_)-expressing MSNs. The remaining 5% of striatal neurons are comprised of a variety of cholinergic and GABAergic interneurons (Kawaguchi et al., 1995, Mallet et al., 2005), which can locally control the activity of the MSNs. The D_1_ and D_2_-expressing MSNs are the projection neurons of the striatum and give rise to the so-called direct and indirect pathways, respectively (Gerfen et al., 1990, Smith et al., 1998) depending on their projections to downstream nuclei of the basal ganglia.

The direct pathway MSNs project directly to the output nuclei of the basal ganglia; the *globus pallidus internus* (GPi) and *substantia nigra pars reticulata* (SNr). These two nuclei consist of tonically active GABAergic neurons whose main target are the glutamatergic neurons of the motor thalamus. The activity of the striatal GABAergic D_1_ MSNs will inhibit the activity of the GPi and SNr neurons and in effect allow a release of their GABAergic control of thalamic activity, which is then able to activate the motor cortex neurons and facilitate movement. Conversely the indirect MSNs project to the GPi and SNr indirectly as they make their synapses first with the GABAergic neurons of the *globus pallidus externus* (GPe). GPe projects to the subthalamic nucleus, which then projects to GPi and SNr as well as sending projections directly to the SNr and GPi. In effect the activity of striatal GABAergic D_2_ MSNs will inhibit the tonically active neurons of the GPe. The reduced activity of the GABAergic neurons of the GPe will allow for an increased activity of GPi and SNr neurons and a stronger inhibitory control of thalamic neurons (Fig. 1A). Although this is an oversimplification, it is thought that through balanced activity in these two pathways; the direct D_1_ pathway facilitating movement and the indirect D_2_ pathway inhibiting movement the striatum can exert control on motor behaviour (Kravitz et al., 2010, Gerfen and Surmeier, 2011).

The activity of the MSNs is not only determined by excitatory glutamatergic input but further controlled by local GABAergic and cholinergic interneurons which make up the remaining 5% of striatal neurons (Kawaguchi et al., 1995, Mallet et al., 2005). MSNs also regulate each other through reciprocal inhibitory connections (Plenz, 2003). Lastly, a large number of neuromodulators including histamine (Haas and Panula, 2003, Ellender et al., 2011) and dopamine (Schulz, 2002, Ungless, 2004, Surmeier et al., 2007), among others (Aston-Jones and Bloom, 1981, Steinbusch, 1981, Mathur et al., 2011, Parent et al., 2011), can control the activity of this circuit in complicated ways. The combined excitatory drive to the MSNs and the neuromodulatory state of the striatum will determine if and when the MSNs fire action potentials and the balance in activity between these two pathways will differentially affect motor behaviour (Kravitz et al., 2010).

To understand how neuromodulators can control the activity of complex circuits such as the basal ganglia we need to understand when, where and how they exert their effect. We need to know when histamine is released, where histamine can act, i.e. which brain regions and neurons express histamine receptors, and how this affects the electrical properties of the neurons and by extension the circuit they comprise. There is now strong evidence that histamine exerts control on the activity of the striatum and as such the behaviour of the basal ganglia as a whole, which we will discuss in the next section.

## 3 Histamine and the striatum

### 3.1 Diurnal release of histamine in the striatum

The histaminergic neurons in the TMN of the hypothalamus fire at high frequency during wakefulness (Takahashi et al., 2006, Saper et al., 2010, Lin et al., 2011) and are virtually silent during sleep (Haas and Panula, 2003, Takahashi et al., 2006, Takahashi et al., 2010, Sakai et al., 2010). The histaminergic neurons project to nearly all parts of the brain (Watanabe et al., 1983, Haas and Panula, 2003) including a projection to the striatum (Fig. 1B). The release of histamine occurs at both the dendritic and axonal arbour through synaptic and non-synaptic release mechanisms (Cumming et al., 1991) and exhibits a diurnal release in many brain structures (Mochizuki et al., 1992, Chu et al., 2004, Zant et al., 2012) most likely including the striatum (Cumming et al., 1991, Adachi et al., 1992, Yoshitake et al., 2003, Castellan Baldan et al., 2014), although complete 24-h microdialysis experiments have yet to be explicitly done for this structure. Thus, it is likely that histamine can exert a diurnal control on the activity of the basal ganglia circuitry. Not only does the activity of histaminergic neurons follow a circadian rhythm it has recently been shown that the mRNA levels of the histamine synthesizing enzyme *histidine decarboxylase* (HDC) also exhibit a diurnal rhythm (Yu et al., 2014) emphasizing a tight diurnal synergy between neural activity and gene expression in this structure. Nevertheless, histaminergic neurons can become active and release histamine immediately and independent of the time of waking (Zant et al., 2012), which suggests that the amount of histamine synthesized and released might well be different when wakefulness occurs outside of the normal circadian rhythm (Yu et al., 2014). Within periods of wakefulness histaminergic neurons further regulate their activity, such that they are highly active during periods of increased attention (Takahashi et al., 2006) and certain motivated behaviours (Torrealba et al., 2012). Although the histaminergic neurons of the TMN are the only neurons producing histamine in the adult brain, other neurons have been shown to express the synthesizing enzyme HDC (Zecharia et al., 2012) or histamine (Vanhala et al., 1994) transiently early in development. Indeed mast cells might also be a further source of striatal histamine (Russell et al., 1990, Watanabe and Wada, 1991).

To date no specific histamine transporters or uptake mechanisms have been described in the brain, although low affinity organic cation transporters might play a role in the uptake of histamine (Amphoux et al., 2006). Thus, histamine is considered to be inactivated in the extracellular space by *histamine methyltransferase* (HMT) with a half-life ranging in the tens of minutes (Pollard et al., 1974, Cumming et al., 1991, Schwartz et al., 1991). This suggests that histamine released by tonically active histaminergic neurons during periods of wakefulness (Reiner and McGeer, 1987, Steininger et al., 1999, Takahashi et al., 2006) may diffuse from the site of release and affect large, if not all, areas of the striatum. Although shown immunohistochemically (Vincent et al., 1983) a recent study demonstrated that a subset of TMN neurons can also co-release the neurotransmitter GABA which can act in a comparable paracrine fashion (Yu et al., 2015), affecting both the electrical properties of striatal neurons and motor behaviour, suggesting that both may act in concert across large areas of the striatum.

### 3.2 Striatal expression of histamine receptors

The high expression of histamine receptors in the striatum (Hill and Young, 1980, Martinez-Mir et al., 1990, Vizuete et al., 1997, Pillot et al., 2002) supports the notion of a widespread effect of histamine on striatal function. To date three main types of histamine receptors (H_1_, H_2_ and H_3_ receptors), all G-protein coupled receptors (GPCRs), have been described in the striatum, which can respond to the released histamine (Haas and Panula, 2003). The H_1_ receptor (Hill and Young, 1980, Yamashita et al., 1991) and H_2_ receptor (Gantz et al., 1991, Traiffort et al., 1992) are mainly postsynaptic receptors positively coupled to intracellular pathways controlling excitability and indeed their activation has mostly excitatory actions on neurons (Schwartz et al., 1991, Haas and Panula, 2003). The H_3_ receptor is mainly thought to be a presynaptic receptor regulating neurotransmitter release through the negative modulation of Ca^2+^ inflow through voltage-activated Ca^2+^ channels (Brown and Haas, 1999), but postsynaptic effects cannot be excluded (Ellenbroek and Ghiabi, 2014). The H_3_ receptor (Lovenberg et al., 1999) is especially highly expressed in the striatum as shown in a series of studies (Pollard et al., 1993, Cumming and Gjedde, 1994, Goodchild et al., 1999, Pillot et al., 2002, González-Sepúlveda et al., 2013). Lastly, the recently described H_4_ receptor seems to be highly expressed by cells of hematopoietic origin and preliminary evidence suggests it might be expressed in the CNS also (Connelly et al., 2009, Strakhova et al., 2009), but this needs further study. Several excellent recent reviews on histaminergic receptors can be found in this special issue and elsewhere (Leurs et al., 2009, Panula et al., 2015).

### 3.3 Functional evidence of histaminergic modulation of striatal circuits

One of the first suggestions that histamine might affect the circuits of the basal ganglia was detailed in a publication in the journal Neuropharmacology nearly 40 years ago (Nowak et al., 1977). In this study the authors observed that injection of histamine in the lateral ventricles of rats induced hypokinesia or akinesia, which was discussed in the context of histaminergic modulation of the cholinergic system of the basal ganglia. A similar decrease in motor behaviour, but followed by a transient increase in locomotion, was later seen after direct injections of histamine in the striatum (Bristow and Bennett, 1988). Already shown for other brain regions (Haas and Konnerth, 1983, McCormick and Williamson, 1991), the earliest functional evidence that histamine directly affected striatal neurons consists of the observation that histamine can modulate the intrinsic electrical properties of striatal cholinergic interneurons (Munakata and Akaike, 1994). In this study the authors performed perforated patch-clamp electrophysiological recordings from dissociated rat striatal cholinergic interneurons and found that a range of concentrations of histamine can depolarize cholinergic neurons by acting at H_1_ and H_2_ receptors and reducing an outward K^+^ current. This depolarizing effect was later confirmed by subsequent *in vitro* studies in acute striatal slices (Bell et al., 2000) and *in vivo* studies showing an increase in acetylcholine release upon microdialysis of histamine (Prast et al., 1999a, Prast et al., 1999b), although the latter authors suggest there might be an indirect way to activate cholinergic interneurons through actions of histamine at dopaminergic afferents. That histamine can also have inhibitory effects on striatal circuits was shown in subsequent studies. For example, it was shown that histamine can negatively regulate the release of GABA from MSNs by acting at H_3_ receptors (Garcia et al., 1997) in acute SNr slices containing the MSN striatonigral terminals, as well as in acute striatal slices (Arias-Montano et al., 2001). Subsequent studies showed that histamine could also reduce the release of glutamate from striatal synaptosomes (Molina-Hernandez et al., 2001), as well as reduce the electrically evoked glutamatergic field responses in corticostriatal slices (Doreulee et al., 2001, Sergeeva et al., 2005), both by acting at H_3_ receptors.

It was thus clear that histamine could regulate some aspects of the striatal circuit but there were still many questions outstanding. These included the potential modulation of the thalamic projection to the striatum, which forms the other major excitatory glutamatergic afferent to the striatum (Smith et al., 2014), and the potential differential modulation of the two main types of striatal MSN (see Table 1) Indeed, several *in vivo* studies of the activity patterns in the striatum came to different conclusions on the effect of histamine on striatal neural activity with both increases (Sittig and Davidowa, 2001) and decreases (Chronister et al., 1982) in neural activity reported upon histamine infusion *in vivo*. Most likely these conflicting results result from the inherent heterogeneity of striatal neurons. The aim of our recent study was therefore to investigate the histaminergic modulation of the striatum using an *in vitro* slice preparation where we could control for this heterogeneity using *post hoc* morphological and immunohistochemical identification of recorded cells (Ellender et al., 2011) as well as including all main striatal glutamatergic and GABAergic afferents. Future studies of the effect of histamine on the striatal circuit activity *in vivo* will likely benefit from similar techniques allowing for *post hoc* anatomical classification of neuron type (Huerta-Ocampo et al., 2014).

In our study we investigated the effect of 10 μM histamine application on striatal synaptic transmission in acute brain slices of adult mice. Although this concentration is effective to study histamine *in vitro* (Brown and Haas, 1999, Atzori et al., 2000, Doreulee et al., 2001) it must be kept in mind that histamine concentrations in the intact brain might well be lower (Yoshitake, 2003, Chu et al., 2004). Deciding on the correct concentration is a general problem with *in vitro* preparations, as it is difficult to predict what the final concentration within the tissue will be. Moreover, acute striatal slices were generated in the morning of each day, corresponding to the dormant stage for mice, which could well affect the neuronal responses to histamine application as histamine receptor membrane expression might also be under diurnal regulation analogous to other receptors (Karatsoreos et al., 2006).

We first investigated the effect of histamine on corticostriatal transmission and the membrane voltage of recorded MSNs. Viral delivery of the light-activatable channel channelrhodopsin-2 (ChR2) (Zhang et al., 2006) in the cortex of CAMKII-cre mice allowed for the expression and subsequent light activation of cortical excitatory neurons. We find that both electrical stimulation of the cortical fibres in the external capsule or optogenetic activation of cortical fibres could elicit glutamatergic EPSPs in recorded MSNs which were reduced in amplitude by histamine to a similar degree (by approximately 20–40%) for both D_1_ and D_2_-expressing MSNs. Co-application of histamine and the H_3_ receptor antagonist thioperamide abolished this effect suggesting that histamine acts at the H_3_ receptors to negatively modulate cortical glutamatergic transmission, consistent with previous observations (Doreulee et al., 2001). Both types of MSN significantly depolarized in the presence of histamine, by approximately 7 mV, through activation of H_2_ receptors as this effect could be blocked by the antagonist ranitidine.

Next, we investigated the effect of histamine on the second major excitatory glutamatergic afferent pathway to striatal MSNs coming from the thalamus and specifically the intralaminar thalamic nuclei (Groenewegen and Berendse, 1994, Lacey et al., 2007, Ellender et al., 2013, Smith et al., 2014). We used an optogenetic approach to improve the isolation of thalamic afferents to the striatum and avoid erroneous activation of passing fibres or disynaptic activation (thalamo-cortico-striatal), which could result from using conventional electrical stimulation. We found that histamine reduces the thalamic excitatory input to both D_1_ and D_2_-expressing MSNs (by approximately 60%) and this also depended on the H_3_ receptor. This is the first observation that histamine strongly controls the glutamatergic release from thalamic fibres in the striatum. Moreover, a direct histaminergic modulation of the thalamus itself is also likely as the thalamus exhibits high levels of H_3_ receptors (Pollard et al., 1993). Indeed it has been shown that the activity pattern of thalamic neurons can be modulated by infusion of histamine *in vivo* altering their pattern of firing (Sittig and Davidowa, 2001). The fact that the histaminergic system, important for the maintenance of arousal and vigilance (Lin et al., 1988, Parmentier et al., 2002) can directly modulate the intralaminar thalamic nuclei and thalamostriatal synapses fits with the idea that the intralaminar nuclei have a general activating role in multiple brain regions (Groenewegen and Berendse, 1994, Kinomura et al., 1996, Matsumoto et al., 2001).

To investigate if the observed histaminergic modulation of synaptic transmission occurred presynaptically on cortical and thalamic afferents we performed paired-pulse stimulation experiments. These consisted of two sequential stimulations, given either electrically or optically in close succession, which can reveal if synapses exhibit short-term facilitation (postsynaptic responses which sequentially increase in amplitude) or short-term depression (postsynaptic responses which sequentially decrease in amplitude) of their baseline responses (Thomson, 2000). They can reveal if neuromodulators such as histamine act presynaptically as this would likely affect the paired-pulse ratio at these synapses. We found that histamine significantly modulates the paired-pulse ratio at both cortical (see also González-Sepúlveda et al., 2013 and Doreulee et al., 2001) and thalamic afferents on both types of MSN, which could be blocked by application of the H_3_ receptor antagonist thioperamide. Histamine altered the paired-pulse ratio at thalamic synapses to such an extent that they converted from depressing to facilitating synapses. This suggests that thalamic inputs are selectively facilitated over cortical inputs during periods of histaminergic innervation i.e. during wakefulness. It is important to realize that the use of optogenetic tools are not without confounds. As ChR2 is calcium permeable this could alter the short-term plastic properties of synapses. Indeed, it was recently shown that the short-term plastic properties of a host of synapses differed whether electrical or optogenetic stimulation was used and even differed depending on the serotype of adeno-associated virus (AAV) used for ChR2 plasmid delivery (Jackman et al., 2014). Although thalamostriatal synapses were not included in this study it would suggest that the modulatory role of histamine on thalamostriatal synapses should be revisited using different methodologies, e.g. using conventional electrical stimulation in brain slices cut at a particular angle to retain the thalamostriatal afferents (Smeal et al., 2007, Smeal et al., 2008, Ding et al., 2008).

Next, we investigated to what extent striatal GABAergic transmission was modulated by histamine. As a first approach we investigated bulk GABAergic transmission in the striatum by placing an electrical stimulating electrode in the striatum to activate all GABAergic afferents simultaneously; likely originating from local MSNs (Plenz, 2003) and interneurons (Kawaguchi et al., 1995, Mallet et al., 2005) as well as from extrastriatal locations such as the GP (Mallet et al., 2012). We found that bulk GABAergic transmission is significantly reduced in the presence of histamine (by approximately 50%). This mainly seems to be the result of H_3_ receptor activation, consistent with previous observations (Arias-Montano et al., 2001), although for the direct pathway D_1_-expressing MSNs part of this negative modulation seems to be the result of histamine acting at H_2_ receptors. This latter result could be explained by histamine acting at the cholinergic interneurons of the striatum (Munakata and Akaike, 1994, Bell et al., 2000) with the released acetylcholine acting at the GABergic synapses on D_1_-expressing MSNs (Sugita et al., 1991, Koos and Tepper, 2002), although other neuromodulatory afferents might well be involved (Prast et al., 1999a, Threlfell et al., 2012). Indeed, a prominent role for histamine in modulating cholinergic neurons was recently reported for the basal forebrain (Zant et al., 2012) and suggested in mouse models of l-DOPA induced dyskinesia (Lim et al., 2015). We did not investigate if histamine co-applied with both H_2_ and H_3_ antagonists simultaneously fully abolished the histamine-induced reduction in GABA release, which leaves open the possibility for a role for the H_1_ receptor in modulating striatal GABAergic transmission.

Lastly, to isolate specific types of GABAergic synapses we performed simultaneous patch-clamp recordings from pairs of striatal neurons. We performed paired recordings from parvalbumin-positive fast-spiking interneurons and MSNs (Kawaguchi et al., 1995). Parvalbumin-positive fast-spiking interneurons generate feedforward GABAergic inhibition of MSNs (Mallet et al., 2005) and due to the location of their synapses close to the soma of MSNs are ideally placed to shape MSN spike timing (Koos and Tepper, 1999). We also performed recordings from pairs of MSNs to investigate the reciprocal connections between MSNs in the striatum. Reciprocal connections between MSNs mediate lateral feedback inhibition, which can either be feedback-facilitatory or feedback-inhibitory (Plenz, 2003). Due to the location of their synapses across the extent of the MSN dendritic arbour they are ideally suited to control the integration of cortical and thalamic excitatory inputs on the MSNs (Plenz, 2003). We found that histamine only negatively modulates the reciprocal connections between MSNs and not the connections between fast-spiking interneurons onto MSNs. It is likely this is mediated through H_3_ receptor activation, as shown for bulk GABAergic transmission, but we did not investigate this explicitly.

In conclusion, we suggest that histamine dynamically modulates many aspects of the functional connectivity within the striatum. Firstly, histamine suppresses both the cortical and thalamic excitatory drive to MSNs by acting at presynaptic H_3_ receptors. Secondly, histamine selectively modulates the dynamics of thalamostriatal, but not corticostriatal, synapses leading to a facilitation of thalamic input. Thirdly, histamine suppresses lateral feedback inhibition between MSNs without affecting feedforward inhibition mediated by parvalbumin-positive fast-spiking interneurons onto MSNs. These findings suggest that when histaminergic neurons are active, such as during wakefulness and periods of increased attention, the striatum will have a facilitated response to thalamostriatal input and be dominated by feedforward inhibition. Although shown to exist anatomically the lateral inhibitory connections between MSNs were initially thought to be weak or non-functional (Jaeger et al., 1994). More recently they have not only been shown to be functional but also rather abundant with connectivity measures ranging from 10% (e.g. 1 functional connection in 10 putative connections as tested in 5 pairs of MSNs) (Tunstall et al., 2002), 15% (Planert et al., 2010) to 30% (Taverna et al., 2008), or even as high as 63% (Chuhma et al., 2011) connectivity. These recent observations suggest that GABAergic synaptic connections between MSNs are exceedingly well placed to shape normal striatal activity (Plenz, 2003), and by extent control motor system output and behaviour (Mink, 1996, Redgrave et al., 1999). Indeed, striatal disinhibition has been shown to generate motor tics in rodents (Bronfeld et al., 2013) and monkeys (McCairn et al., 2009), which would be consistent with this hypothesis. Our recent study would suggest that histamine could tightly regulate this network of lateral inhibitory connections.

What is still largely unknown, and is essential for a complete understanding of the role of histamine on striatal function is, firstly, the modulation of other types of striatal interneurons by histamine (Kawaguchi et al., 1995, Sharott et al., 2012). Secondly, the direct modulation of activity in the afferent structures themselves i.e. cortex (Zant et al., 2012) and thalamus (Groenewegen and Berendse, 1994, Lacey et al., 2007, Ellender et al., 2013, Smith et al., 2014, Varela, 2014), although recent investigations are beginning to address this (Yu et al., 2015). Thirdly, the histaminergic modulation of other striatal neuromodulatory pathways, such as the noradrenergic (Schlicker et al., 1992) and serotoninergic pathways (Dere et al., 2004, Threlfell et al., 2004), which would allow for complex interactions amongst neuromodulators (Threlfell and Cragg, 2011) as suggested for the dopaminergic pathway (see next section). Lastly, it is largely unknown if histamine modulates other basal ganglia structures and downstream motor regions (Chen et al., 2003, Chen et al., 2005) although their widespread projections (Watanabe et al., 1983, Panula et al., 1984) would suggest so. Although it is possible to dissect the differential effects of histamine on all these individual structures and pathways in isolation a powerful approach to investigate histaminergic control of basal ganglia behaviour would combine behavioural analysis and the recording of anatomically identified neurons (Lapray et al., 2012) in conjunction with optogenetic technologies to stimulate histaminergic afferents (Yu et al., 2015). This has become technically feasible with the recent advent of suitable HDC-cre (Yanovsky et al., 2012, Zecharia et al., 2012) and optogenetic mouse lines (Madisen et al., 2012). Nonetheless, it is already apparent from current *in vivo* studies characterizing the detailed behaviour of histamine-deficient HDC knockout animals that they exhibit symptoms suggesting basal ganglia dysfunction (Castellan Baldan et al., 2014) as well as problems regarding wakefulness (Lin et al., 1988, Parmentier et al., 2002, Anaclet et al., 2009). Indeed, a recently described patient cohort exhibited both reduced histamine synthesis and aberrant motor behaviour (Ercan-Sencicek et al., 2010), suggesting a central role for histamine in controlling normal basal ganglia function and an aberrant histaminergic system in basal ganglia disorders.

## 4 Histamine and striatal disorders

The striatum is the site of several devastating neurological disorders and altered striatal function has been implicated in Parkinson's disease and Huntington's disease, which we will discuss below in the context of the histaminergic system, as well as schizophrenia and addiction amongst others (Simpson et al., 2010, Graybiel, 2000, Kreitzer and Malenka, 2008, Robbins et al., 2008).

### 4.1 Parkinson's disease

Several studies have used post-mortem brain tissue of Parkinson's disease patients to investigate potential alterations in the histaminergic system and the basal ganglia. The number of histaminergic neurons of the TMN (Nakamura et al., 1996) and HDC activity (Garbarg et al., 1983) appear unaltered in Parkinson's disease. However, levels of histamine are markedly increased in the striatum, SNr and GP of Parkinson's disease patients (Rinne et al., 2002). A similar increase in the levels of histamine, concomitant with an increase in H_3_ receptor mRNA expression and H_3_ receptor radioligand binding, was shown for a second independent group of Parkinson's disease patients (Anichtchik et al., 2001), as well as in the 6-hydroxydopamine (6-OHDA) model of Parkinson's disease in rats (Ryu et al., 1994, Anichtchik et al., 2000). However, other studies have found little to no change in H_3_ receptor expression or radioligand binding in human Parkinson's disease patients (Goodchild et al., 1999, Shan et al., 2012) suggesting further investigation is needed. Indeed, Goodchild et al. found a significant reduction in H_3_ receptor radioligand binding in tissue of Huntington's disease, but not Parkinson's disease, patients. Some of these discrepancies could result from the different methodologies employed, for example RT-PCR (Shan et al., 2012) and radioligand binding (Goodchild et al., 1999). Lastly, although the levels of histamine were shown to be increased (Anichtchik et al., 2001, Rinne et al., 2002) the levels of transcripts for the metabolizing enzyme HMT have also been shown to be increased (Shan et al., 2012) suggesting compensatory mechanisms might be in place.

Some of the findings might result from specific treatment programs for the various Parkinson's disease patient groups, such as treatment with dopaminergic precursors such as L-DOPA. A close link between the histaminergic and dopaminergic system is likely as histaminergic neurons in the TMN also express the dopamine-synthesizing enzyme *DOPA decarboxylase* (DDC). This, in combination with their expression of *vesicular monoamine transporter type 2* (VMAT2), which allows them to take-up L-DOPA, suggest that they might co-release dopamine and histamine in certain circumstances, such as when Parkinson's patients are treated with L-DOPA (Yanovsky et al., 2011). Histamine has been shown to negatively regulate the release of dopamine in the striatum (Schlicker et al., 1993, Nowak et al., 2008, Castellan Baldan et al., 2014) consistent with the observation of an increased dopaminergic tone in HDC-KO mice (Rapanelli et al., 2014). Lastly, a direct interaction between histaminergic H_3_ receptors and dopamine receptors has been shown to occur in co-expressing culture systems (Ferrada et al., 2009) and potentially to occur also *in vivo* (Moreno et al., 2011). Together these lines of evidence suggest a potential synergy between the histaminergic and dopaminergic systems in Parkinson's disease which warrants further investigation (Table 1).

### 4.2 Tourette's syndrome

Recent evidence has implicated a specific dysfunction of the histaminergic system in the symptoms of Tourette's syndrome (Ercan-Sencicek et al., 2010). Tourette's syndrome involves uncontrollable motor and vocal tics in which the dysfunction of the cortico-striato-thalamo-cortical recurrent pathway is thought to play a major role (Jeffries et al., 2002). We will discuss this finding in the context of our recent observations of histaminergic control of basal ganglia circuits and the idea that reduced levels of histamine might facilitate crosstalk between the normally segregated pathways of the cortico-striato-thalamo-cortico loop.

The study of Ercan-Sencicek and colleagues describes a linkage study of a family in which all children developed Tourette's syndrome. Through linkage analysis the authors find a mutation on chromosome 15 in the gene encoding for the histamine-synthesizing enzyme HDC in the father only. This mutation consisted of a G to A base mutation leading to a W317X, tryptophan to missense, transition in exon 9 of the HDC gene resulting in a premature stop codon resulting in a truncation of the HDC protein. This truncated protein (a 35 kD instead of the normal 54 kD protein) misses key segments of the active domain and acts as a dominant negative mutation as it negatively affects the enzymatic activity of any remaining healthy copies. It is thought to be a rare mutation, as it was not found in the wider population or indeed in a second independent large cohort of Tourette's syndrome patients. Nonetheless, it points to a clear role for histaminergic transmission in the mechanism and modulation of Tourette's syndrome and tics. Indeed, similar discoveries of rare genetic disorders in Parkinson's disease such as mutations in the ɑ-synuclein gene (Mezey et al., 1998) and in Alzheimer's disease in the amyloid precursor protein gene (Chartier-Harlin et al., 1991, Goate et al., 1991, Murrell et al., 1991) have enabled studies leading to detailed insight in disease aetiology.

To gain mechanistic insight a recent comparative study of HDC gene function was performed in wildtype, heterozygous (HET) and KO animals for the HDC gene (Castellan Baldan et al., 2014). In this study the authors confirmed that in HDC transgenic mice, as suggested for Tourette's syndrome patients, brain histamine concentrations were significantly reduced. The mice had a gene-dose dependent decrease in histamine concentration in the hypothalamus, striatum and cortex in both the HET and KO mice suggesting haploinsufficiency. Although the general baseline behaviour of the transgenic mice appeared normal they exhibited a decrease in pre-pulse inhibition of startle responses. This was also observed in the W317X patient cohort and is a hallmark of deficits or abnormalities of sensorimotor gating. Moreover, whereas WT mice responded with increased locomotion upon amphetamine administration, both HET and KO animals responded with an increase in a range of stereotypies such as repetitive focussed sniffing and orofacial movements reminiscent of the symptoms found in Tourette's syndrome patients. This behavioural phenotype could be attenuated by pre-treatment with the D_2_ receptor antagonist, haloperidol, or infusion with histamine. Histamine infusion likely reduces the concentration of dopamine in the striatum by acting at H_3_ receptors on dopaminergic afferents (Schlicker et al., 1993). Indeed, Castellan Baldan and colleagues find that dopamine levels are increased in HDC KO mice. The chronic increased levels of dopamine could lead to alterations in the expression of dopamine receptors and indeed they find that the levels of D_2_/D_3_ receptor expression in the SNr and pallidum of W317X patient and KO mice are increased. Although changes were observed in the striatum they were modest. In conclusion, these studies suggest that reduced histamine production can produce symptoms of Tourette's syndrome potentially through dysregulation of dopaminergic modulation of the basal ganglia and further emphasizes a functional association between the histaminergic and dopaminergic systems (Ferrada et al., 2009, Moreno et al., 2011, Yanovsky et al., 2011, Rapanelli et al., 2014).

A powerful concept guiding basal ganglia research has been the idea of parallel cortico-striato-thalamo-cortical loops specialized for the processing of different types of behaviourally relevant information (Alexander et al., 1986, Haber and Knutson, 2010, Choi et al., 2012). It has been suggested that the degree of interaction between competing excitatory inputs needs to be tightly regulated as insufficient inhibition of competing pathways can lead to erroneous activation of unwanted actions, seen for example as motor and verbal tics, such as those seen in Tourette's syndrome. Conversely, overriding inhibition of competing pathways can hinder behavioural or attentional switching as seen in addictive compulsions and obsessive-compulsive disorders (Redgrave et al., 1999). Such crosstalk could arise within or between any of the different basal ganglia nuclei including the striatum. In the striatum the GABAergic MSNs account for the majority of neurons and the majority of all synaptic connections in the striatum consist of the lateral GABAergic inhibitory connections between MSNs. Our recent observation of strong histaminergic control of this network of lateral feedback inhibitory connections between MSNs (Ellender et al., 2011) and observations of altered striatal histaminergic tone in Tourette's syndrome (Castellan Baldan et al., 2014) could suggest that this might well be the site at which dysfunctional crosstalk between normally segregated pathways could occur. Indeed, alterations in the histaminergic and GABAergic systems (Kalanithi et al., 2005, Kataoka et al., 2010) are thought to be common features in tic disorders (Fernandez et al., 2012).

## 5 Conclusion

These are exciting times for the study of the neuromodulator histamine and its role in regulating neural circuit activity. Increasing evidence points to a role for histaminergic modulation of basal ganglia and striatal function. The finding that histaminergic dysfunction is causally related to Tourette's syndrome and histaminergic supplementation alleviates part of the symptoms warrants investigation whether other tic disorders might also benefit from histaminergic treatment. Indeed, investigations of the therapeutic potential of various drugs acting at histamine receptors (Huotari et al., 2000, Gomez-Ramirez et al., 2006, Kuhne et al., 2011, Passani and Blandina, 2011) in either treating neurological disorders or enhancing treatment efficacy (Johnston et al., 2010) are ongoing. The development of new transgenic mouse lines, such as the HDC-cre lines (Yanovsky et al., 2012, Zecharia et al., 2012) and histamine receptor KO lines (Toyota et al., 2002) will prove invaluable for future research. Lastly, observations of heterogeneity of the histaminergic neurons in the TMN (Blandina et al., 2012, Giannoni et al., 2009, Williams et al., 2014, Yu et al., 2015) in co-expression of other neurotransmitters, receptor expression and distinct projection targets, suggests complexity which will need to be taken into account if we are to understand how histamine controls various neural circuits and behaviour.

## Figures and Tables

**Table 1:** Histaminergic modulation of striatal afferents and striatal neurons.

	Experimental design	Effect of histamine	Receptors	Reference
*Glutamate*				
Cortex	Electrical stimulation of cortical afferents and striatal field recordings *in vitro*	Decrease release	H3	Doreulee et al. (2001)
4-AP induced glutamate release from synaptosomes *in vitro*	Decrease release	H3	Molina-Hernandez et al. (2001)
Electrical stimulation of cortical afferents and striatal field recordings *in vitro*	Decrease release	?	Sergeeva et al. (2005)
Electrical and ChR2-mediated optical stimulation of cortical afferents in striatal slices	Decrease release	H3	Ellender et al. (2011)
Electrical stimulation of cortical afferents and striatal field recordings in striatal slices	Decrease release	H3	González-Sepulveda et al. (2013)
Thalamus	4-AP induced glutamate release from synaptosomes *in vitro*	Decrease release	H3	Molina-Hernandez et al. (2001)
ChR2-mediated optical activation of thalamic afferents in striatal slices	Decrease release	H3	Ellender et al. (2011)
*GABA*				
MSNs	High [K+] and D1 agonist induced [3H]GABA release in SNr slices	Decrease release	H3	Garcia et al. (1997)
Paired whole-cell patch-clamp recordings in striatal slices	Decrease release	?	Ellender et al. (2011)
Parvalbumin interneurons – MSNs	Paired whole-cell patch-clamp recordings in striatal slices	No change	–	Ellender et al. (2011)
Other GABAergic sources	High [K+] and D1 agonist induced [3H]GABA release in striatal slices	Decrease release	H3	Arias-Montana et al. (2001)
Intrastriatal electrical stimulation evoked bulk GABA release in striatal slices	Decrease release	H3 and H2	Ellender et al. (2011)
*Cholinergic interneurons*				
	High [K+] activation of disscociated Cholinergic interneurons	Depolarization	H1 and H2	Munakata and Akaike (1994)
	Histamine induced acetylcholine release in striatum *in vivo*	Increase release	H3	Prast et al. (1999b)
	Whole-cell patch-clamp of ChI in striatal slices	Depolarization	H1	Bell et al. (2000)
*Dopamine*				
	Electrical stimulation of [3H]dopamine release in striatal slices	Decrease release	H3	Schlicker et al. (1993)
	Generation of [3H]dopamine in striatal tissue	Reduced synthesis	H3	González-Sepulveda et al. (2013)

4-AP: 4-aminopyridine.

ChR2: channelrhodopsin-2.

SNr: substantia nigra pars reticulata.
